# Internet-based support system and rehabilitation of drug users under maintenance treatment

**DOI:** 10.22088/cjim.8.4.335

**Published:** 2017

**Authors:** Saeid Komasi, Mozhgan Saeidi, Ali Soroush

**Affiliations:** 1 Clinical Research Development Center, Imam Reza Hospital, Kermanshah University of Medical Sciences, Kermanshah, Iran.; 2 Cardiac Rehabilitation Center, Imam Ali Hospital, Kermanshah University of Medical Sciences, Kermanshah, Iran.; 3 Lifestyle Modification Research Center, Imam Reza Hospital, Kermanshah University of Medical Sciences, Kermanshah, Iran.

**Keywords:** Drug addiction, Healthcare delivery, Support groups

**Dear Editor,**

In Iran, a daily considerable large number of addicts are under the methadone/buprenorphine maintenance treatment (MMT/BMT) outpatient clinics (1). In these centers, pharmacotherapy with methadone and buprenorphine along with psychotherapy have important roles in modifying thoughts, reducing the tendency for drug abuse and other risky behaviors related to drugs and survival (1, 2). However, lack of adherence to treatment is one of the challenges that is influenced by various factors. One of these factors is the quantity and quality of the patient’s family support during treatment. Family support is associated with an increased physical, mental, environmental and social health (3). Drug abuse history and frequency performing risky behaviors and imposing financial and emotional pressures to family members generally cause their inappropriate behavior reactions. Mistrust and lack of financial and psychological support from patients under treatment are some of the factors that usually facilitate treatment failure. Paying attention to this issue and providing timely and appropriate training to the patient’s family members can be effective in increasing family support, thus resulting in a successful treatment (4).

Extending psychological counseling and training to drug addicts’ family members under MMT/BMT and promoting their supportive motivation may be an effective component in patient recovery. Nonetheless, a large number of patients and time constraints during MMT/BMT weaken the possibility of providing psychological counseling and face-to-face training to the family members. With these circumstances, the use of indirect delivery formats without place and time constraints can be effective in reducing barriers to provide services to families. Furthermore, these methods can be mentioned via interventions delivered online (i.e. Websites) and through mobile phones (5). During recent years, the use of mobile phone and common applications such as telegram, viber, whatsapp messenger, wechat, mobogram, and line messenger has increased dramatically, educational services can be given to the patient’s support system through this format. In other words, MMT/BMT center supervisor and psychologist can provide each patient’s family members audio files and video clips tailored to their patients (sex, type of abused substance, dosage, environmental factors, and etc.) through telegram and other mobile applications. Moreover, the specialists daily or weekly respond to their possible questions. In the service delivery framework format, the physical, mental and medical conditions can be reported through the support system. In addition, using mobile intervention can be reminded of the importance of support system in emergency situations. Although given that the support system plays an important role in healing and preventing treatment failure (3, 4) it is not solely emphasized in the face-to-face delivery rehabilitation services (6), we suggest essential training to be provided in the support system through mobile health interventions. As a result, we recommend using a web-based delivery format and mobile intervention to increase patient – centeredness in the support system and the medical team of MMT/BMT centers.

## Funding/Support:

This study received no grant or funding source.

## Conflict of Interest:

None declared.
